# The Potential of CRISPR/Cas9 to Circumvent the Risk Factor Neurotoxin *β-N-oxalyl-L-α*, *β-diaminopropionic acid* Limiting Wide Acceptance of the Underutilized Grass Pea (*Lathyrus sativus* L.)

**DOI:** 10.3390/cimb46090626

**Published:** 2024-09-21

**Authors:** Abreham Bekele-Alemu, Deribew Girma-Tola, Ayalew Ligaba-Osena

**Affiliations:** 1 Laboratory of Plant Molecular Biology and Biotechnology, Department of Biology, University of North Carolina Greensboro, Greensboro, NC 27412, USA; abalemu@uncg.edu; 2 Department of Biology, College of Natural Sciences, Salale University, Fitche P.O. Box 245, Ethiopia; ofif2010@gmail.com

**Keywords:** grass pea, β-ODAP, β-ODAP synthase, β-cyanoalanine synthase, stress tolerant

## Abstract

Grass pea (*Lathyrus sativus* L.) is a protein-rich crop that is resilient to various abiotic stresses, including drought. However, it is not cultivated widely for human consumption due to the neurotoxin *β-N-oxalyl-L-α*, *β-diaminopropionic acid* (β-ODAP) and its association with *neurolathyrism*. Though some varieties with low β-ODAP have been developed through classical breeding, the β-ODAP content is increasing due to genotype x environment interactions. This review covers grass pea nutritional quality, β-ODAP biosynthesis, mechanism of paralysis, traditional ways to reduce β-ODAP, candidate genes for boosting sulfur-containing amino acids, and the potential and targets of gene editing to reduce β-ODAP content. Recently, two key enzymes (β-ODAP synthase and β-cyanoalanine synthase) have been identified in the biosynthetic pathway of β-ODAP. We proposed four strategies through which the genes encoding these enzymes can be targeted and suppressed using CRISPR/Cas9 gene editing. Compared to its homology in *Medicago truncatula*, the grass pea β-ODAP synthase gene sequence and β-cyanoalanine synthase showed 62.9% and 95% similarity, respectively. The β-ODAP synthase converts the final intermediate L-DAPA into toxic β-ODAP, whist β-cyanoalanine synthase converts O-Acetylserine into β-isoxazolin-5-on-2-yl-alanine. Since grass pea is low in methionine and cysteine amino acids, improvement of these amino acids is also needed to boost its protein content. This review contains useful resources for grass pea improvement while also offering potential gene editing strategies to lower β-ODAP levels.

## 1. Background

Grass pea (*Lathyrus sativus* L.) is a legume crop that belongs to the *Fabaceae* family. It has high adaptability and tolerance to abiotic stresses such as drought, salinity, and poor soil [1,2,3]. It is cultivated in various areas of the world, including Eastern Africa, Eurasia, North America, and South America, for both animal feed and human consumption [4]. It requires an average annual rainfall between 300 and 1500 mm [5]. In South Asia and East Africa, grass pea is cultivated on a total of 1.5 million ha of land, with an annual production of 1.2 million tons [6]. However, this figure might have increased recently as its popularity is increasing in developing countries [7] for its tolerance to severe drought.

The grass pea is a diploid plant species with 2n = 2x = 14 chromosomes [8]. In addition to abiotic factors, it is also resistant to several biotic stresses, such as insects and diseases, and has high protein content for human and animal feed [9]. Furthermore, grass pea is superior in yield and nitrogen fixation compared to other legume crops such as beans, peas, and chickpeas [10]. It has immense potential to be expanded to dry and drought-prone areas with increased salinity or an increased tendency to suffer from biotic stresses [11]. Due to its inherent tolerance to harsh weather conditions, some researchers consider grass pea a survival food in drought times [12] that has saved thousands of lives during droughts in Ethiopia, India, Bangladesh, China, and other developing countries [1]. Though not as commonly cultivated as other crops, grass pea production is increasing, especially in developing countries and drought-affected areas, among which Ethiopia, India, and Bangladesh are major producers [7]. In Ethiopia, grass pea became a crop of choice as an off-season crop because of its higher tolerance to adverse environmental conditions like drought, soil infertility, and waterlogging [13], and more recently due to severe and devastating common bean diseases such as anthracnose, rust, bacterial blight, and halo blight that cause 100%, 85%, 62%, and 45% yield losses, respectively [14].

Crops like grass pea, which tolerate both abiotic and biotic stress, are much needed for global agriculture to ensure nutritional security in the face of climate change [15]. Despite having great economic and nutritional values, grass pea has a major drawback. It contains a major neurotoxic anti-nutritional compound known as β-N-oxalyl-L-α, β di-amino propionic acid (β-ODAP) that causes leg paralysis, which is an irreversible spastic paraparesis of the lower limbs in humans [2,16,17,18]. Studies have indicated that grass pea cultivation is limited to certain countries, mainly due to the concerns of β-ODAP toxicity [19,20], which hinders its large-scale cultivation and utilization for nutritional purposes. Hence, developing grass pea varieties with low seed anti-nutritional factors but with high seed protein content would be used for human consumption and nutritional choice. Therefore, reducing β-ODAP content in seeds of this crop becomes pertinent to ensure that it continues to provide food and nutritional security to low-income communities [2,21].

Since the discovery of neuro-excitatory amino acid β-ODAP [22], several screening strategies have been employed to select grass pea varieties with low β-ODAP. However, β-ODAP toxicity is still a common problem in developing countries due to the absence of varieties that are free from unwanted toxic substances. Advances in molecular genetics have shed light on some key enzymes of the β-ODAP biosynthetic pathway [23,24], opening new avenues for targeted knockout of key genes to eliminate this toxin. In this review, classical approaches used to reduce the biosynthesis of β-ODAP and its level, mechanism of action, and toxicity to humans, as well as permanent and more specific biotechnological approaches that can be applied to reduce or eliminate the β-ODAP are reviewed.

## 2. Nutritional Benefits and Side Effects of Grass Pea

Grass pea is a valuable source of protein for low-income families, contributing to their daily diets and ensuring balanced nutrition. Research has shown that grass pea has great potential as a food crop, providing not only protein but also essential micronutrients for both humans and animals [25]. The protein found in grass pea contains 17 out of the 20 amino acids, with a particularly high lysine content [19,21]. In fact, its protein content (28–49%) is higher than other legumes like chickpeas, peas, faba beans, lupine, and kidney beans [21,26]. Additionally, the protein composition of grass pea seeds consists of albumins (14%), globulins (66%), glutelins (15%), and prolamins (5%) [27]. Studies further suggest that grass pea is particularly advantageous for human consumption, as 58% of its fatty acids are classified as polyunsaturated [28]. Additionally, grass pea seeds are rich in carbohydrates, with up to a content of 58% reported [26].

Grass pea seed contains a distinctive non-protein amino acid called L-homoarginine (hArg) [29]. Studies have demonstrated that hArg is utilized to diminish the production of nitric oxide and plays a pivotal role in the management of cardiovascular ailments, as well as the inhibition of tissue-nonspecific alkaline phosphatase (TNAP), an enzyme that fosters vascular calcification [2,30,31]. Consequently, hArg serves as an endogenous safeguard for cardiovascular and metabolic factors [32]. A recent investigation also revealed that hArg supplementation prevents the enlargement of the left ventricle and preserves systolic function in a model of coronary artery disease [33]. Moreover, hArg is implicated in curtailing the expansion of cancerous tumors [34]. The concentration of hArg in human plasma is approximately 1 to 2 μmol/L [35], and it is believed to be synthesized from arginine and L-lysine by the mitochondrial enzyme arginine: glycine amidino transferase [36]. Therefore, grass pea exhibits potential as an exceptional functional food ingredient [37], provided that its antinutritional factor is minimized.

Grass pea-based dishes are not only nutritious but also delicious, making them popular in various regions, including Europe, Africa, and Asia [2]. In Ethiopia, for example, grass pea is prepared in different ways, such as boiling the whole seeds (nifro), roasting them (kollo), using them in traditional sauces (shiro wot and kik wot), or consuming the green unripe seeds as a snack (eshet) by cattle keepers [16]. However, it is important to note that, like other legumes, grass pea seeds have a low level of methionine, which is crucial for human nutrition and brain function [27]. Despite this limitation, the nutritional benefits and versatility of grass pea make it a valuable addition to the diets of low-income families, providing them with much-needed protein and essential nutrients.

The presence of β-ODAP in grass pea seeds has limited its consumption since high consumption may initiate the development of *neurolathyrism*, a disease that results in the degeneration of pyramidal-tract neurons in the spinal cord [38] and leg cortex [39]. The neurodegenerative process results in a spastic paraparesis of the legs without affecting sensory systems [40]. Such a problem can be observed in communities where the consumption of unprocessed grass pea is common. For instance, over 2000 people were affected in Ethiopia in a single village in 1996 [41,42]. After the end of the Spanish Civil War, approximately 1000 patients developed lathyrism as a result of their excessive and prolonged intake of grass pea [43,44]. The incidence of paralysis mostly occurs during drought season, when younger people eat the green ripe seeds on their way to school or when they are watching cattle. Early studies showed that *neurolathyrism* was caused by the long-term overconsumption of grass pea seeds, containing up to 1% of β-ODAP in the seeds [22].

According to a study conducted by Girma et al. [45], farmers in Ethiopia hold the belief that the consumption of unripe grass pea seeds, particularly when combined with milk and milk products, can potentially worsen the occurrence of paralysis. The study aimed to explore the perceptions of farmers regarding the consumption and toxicity of grass pea, and the findings indicated that farmers in the study area perceive a higher risk of toxicity when consuming grass pea ‘kita’ (softened bread) or porridge with milk, butter, or oil. However, Hoque et al. [46] reached a different conclusion, suggesting that the addition of milk can effectively reduce the toxicity of β-ODAP in grass pea. Nevertheless, further laboratory-based investigations are necessary to validate these claims. Moreover, Ethiopian farmers who have family members affected by β-ODAP toxicity propose that sleeping on grass pea straw overnight during the harvesting process could potentially contribute to paralysis. Additionally, farmers associate lathyrism with exposure to vapors emitted from the cooking of grass pea or when harvesting on a cloudy day [47].

The grass pea toxin exists in α and β isomers [48]. The β-isomer accounts for 99% of the ODAP content and the α isomers are non-toxic [23]. The presence of β-ODAP in seeds as free amino acid, especially in drought-tolerant lines, is believed to be responsible for the irreversible paralysis observed in humans [2], which is more prevalent when grass pea is a main source of diet [49,50]. Studies have suggested that deficiencies of certain sulfur-rich amino acids such as cysteine and methionine increase the neurotoxicity level of ODAP [19,51]. According to Fikre et al. [16], dietary supplementation with methionine and cysteine may significantly lower the risk for *neurolathyrism*. Studies have also indicated that the deprivation of both methionine and cysteine increased the toxicity of l-β-ODAP by 66% [52]. Hence, the development of grass pea rich in these two amino acids can be another way to reduce the toxic effects of grass pea.

## 3. Conventional Improvement for β-ODAP Reduction and Influence of Environmental Factors

**Figure 1:**
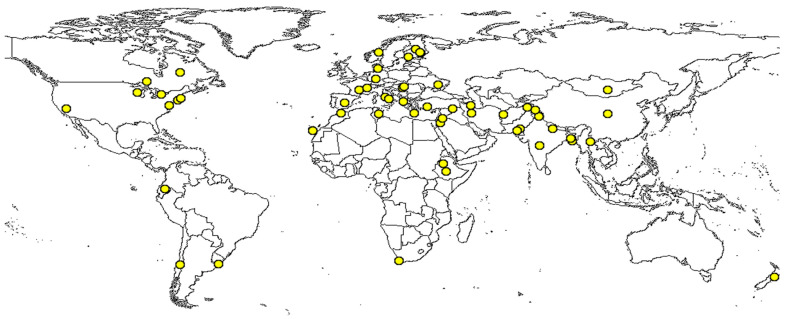
Global specimen registry (yellow circles) of grass pea (*Lathyrus sativus)*. The map was taken from global mapper (https://www.discoverlife.org/mp/20m?act=make_map&kind=Lathyrus+sativus, accessed on 28 March 2024).

The global importance of grass pea as a promising food source has been recognized by Kew’s Millennium Seed Bank [2,3]. Figure 1 Illustrates specimen records of grass pea using global mapper (https://www.discoverlife.org/mp/20m?act=make_map; accessed on 28 March 2024). There is a mild increase in the cultivation of grass pea in some countries, especially in drought-prone regions, due to its remarkable ability to adapt to difficult soil conditions and emerging ecological environments [15]. While toxin-free varieties have yet to be identified, research has indicated that germplasm from South Asia contains relatively high levels of ODAP (0.7–2.4%), whereas those from North Africa, Syria, Turkey, and Cyprus exhibit significantly lower quantities of ODAP (0.02–1.2%) [53]. Despite its ability to thrive in harsh and water-limited environments, only a limited number of scientific approaches have been employed thus far to improve grass pea traits. Through classical screening methods, several grass pea accessions with low ODAP content (0.04–0.1%) have been developed in various parts of the world, including India, Bangladesh, Nepal, Ethiopia, Australia, Canada, Poland, and Turkey [31]. However, it is important to note that this approach may not provide a sustainable and reliable solution to fully address the issue of grass pea toxicity for large-scale production and consumption.

Conventional breeding is centralized fundamentally on the hybridization of selected genotypes followed by the screening and evaluation of the subsequent progenies for the traits of interest. Breeding programs for grass pea improvement are being carried out in several countries, including Australia [54], Bangladesh [55], Canada [56], China [57], Ethiopia [58], India [59], Nepal [60], Syria [53], Poland [28], Italy [61], the USA [62], and Chile [63]. Several varieties and lines have been developed by combining low β-ODAP (<0.1%) with high-yielding and disease-tolerant traits [50,64]. Most of the initial progress in grass pea research aimed at developing low ODAP varieties was made through direct selection from landraces and lines [19].

Various countries have conducted traditional germplasm selection to identify varieties with low ODAP (0.04–0.3%) content. The recommended varieties include Bair Khesari and Bina Khesari in Bangladesh [65,66], Gurbuz-1 in Turkey [67], Clima pink in Nepal [60], and LS 8246 and AC-Greenfix in Canada [56]. In 1966, India introduced the first conventionally enhanced variety called Pusa 24 (P-24), which exhibited a low ODAP content (0.3%) in its seeds [19]. Nonetheless, the instability of the low ODAP trait was a major bottleneck of the P-24 cultivar [68]. In Chile, a cultivar Quila-blanco was also developed to have lower ODAP and higher protein content [69]. In Ethiopia, a low ODAP (0.08%) variety known as Wassie was released in 2006 [70], though its ODAP content was not found to be consistent and has even increased in subsequent field and laboratory tests [47]. Though several germplasm collections and introductions were conventionally screened, varietal performances were found to be inconsistent with ODAP reduction [47], and ODAP content varied from location to location, indicating a strong G × E interaction in determining ODAP content.

Conventional breeding has been unsuccessful in reducing ODAP at a lower level due to the complex inheritance of ODAP, which is greatly influenced by the genotype, environment, and their interactions [19]. An example of this is seen in the variety Wassie, developed in Ethiopia, where the ODAP content has increased from 0.08% to over 0.2% in certain lowland areas [16]. The problem becomes even more severe when the plant is cultivated in a stressed environment [47]. Therefore, recent biotechnological approaches may provide a more dependable strategy to decrease the ODAP content and address the persistent toxicity associated with this compound.

Several studies have shown that various environmental factors, such as drought, salinity, minerals, and heavy metals, affect the biosynthesis of β-ODAP [71,72,73]. The study on the effect of drought, salinity, and deficiency or the oversupply of micronutrients on the biosynthesis of β-ODAP by feeding callus tissue of *Lathyrus sativus* with precursor BIA (β-isoxazolin-5-on-2-yl-alanine) was conducted by Hoque et al. [74]. According to this study, lower concentrations of NaCl decreased the conversion of BIA into β-ODAP, while higher doses of NaCl showed the opposite effects. Mannitol has been reported to increase β-ODAP production, suggesting that drought may increase the biosynthesis of β-ODAP. Furthermore, β-ODAP accumulation in grass pea is thought to be due to the level of total free nitrogenous compounds [19]. According to Jiao et al. [73], the accumulation pattern of β-ODAP in seven-day-old grass pea seedlings grown in a nitrogen-deficient solution was higher as compared to the control. Hence, a nitrogen-poor environment might aggravate β-ODAP content in grass pea. Micronutrients such as Al^3+^, B^3+^, and Co^2+^ have also been reported to increase β-ODAP production, whilst Cu^2+^ and Zn^2+^ played opposite roles [74]. According to Takarz et al. [75], a higher accumulation of β-ODAP was observed in both the shoots and roots of grass pea seedlings under PEG-induced drought stress. This may suggest that β-ODAP or its intermediate product may have a role in drought stress tolerance.

## 4. Biosynthesis of β-ODAP in Grass Pea

**Figure 2:**
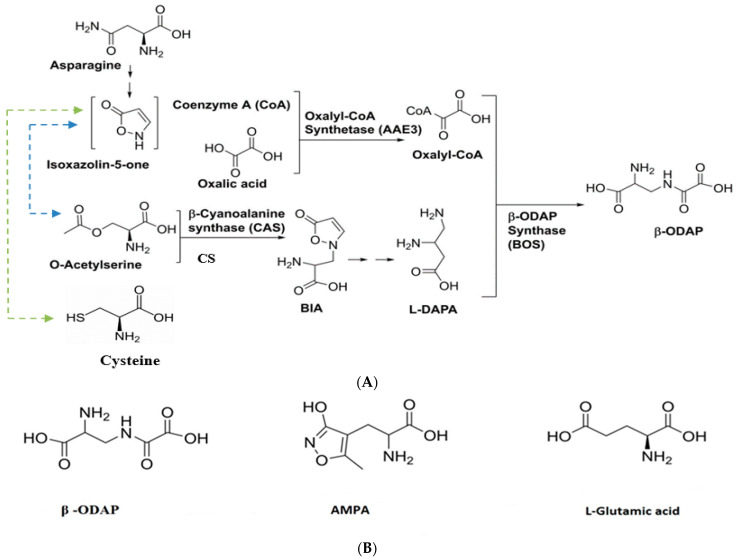
(**A**) Illustration of the proposed biosynthetic pathway of β-ODAP biosynthesis (ChemDraw), adopted from [81]. The product in brackets (Isoxazolin-5-one) is hypothetical and has not been detected in grass pea. BIA is β-(isoxazolin-5-on-2-yl) alanine), a precursor of L-DAPA (L- L-2,3-diaminopropionic acid, a final precursor of β-ODAP). The light green line indicated the formation of BIA from Isoxazolin-5-one and cysteine by the CAS enzyme at high sulfur. The blue line indicates the formation of BIA from Isoxazolin-5-one and O-Acetyl serine at low sulfur by CS enzyme. According to our conserved domain analysis, CAS is a bifunctional enzyme that may also act as CS. (**B**) Structural illustration of L-Glutamic acid analog that competes for AMPA receptor. The structures were drawn using ChemDraw 12.0.

Despite the discovery of β-ODAP’s association with *neurolathyrism* 50 years ago, a complete understanding of its biosynthetic pathway remains elusive. Nevertheless, researchers have made progress in unraveling certain crucial steps in the biosynthesis of β-ODAP by using enzyme extract [76,77,78] and radioisotope studies [79]. The biosynthesis of β-ODAP is initiated with the formation of BIA from isoxazoline-5-one and cysteine, facilitated by the enzyme β-cyanoalanine synthase (CAS) under conditions of high sulfur [51]. However, under low-sulfur conditions, BIA is primarily synthesized from isoxazoline-5-one and O-acetyl-serine, catalyzed by cysteine synthase (CS) [78]. According to the previous study, a gene encoding CAS and at least four other genes encoding CS have been identified from grass pea, and their activities have been characterized [80]. Both CAS and CS are important enzymes in β-ODAP synthesis. Studies by Song et al. [51] indicated that β-Cyanoalanine synthase regulates the accumulation of β-ODAP through interaction with serine acetyltransferase. An attempt to characterize the activity of the genes encoding CAS and CS was recently reported [80]. Figure 2 shows a proposed biosynthetic pathway of β-ODAP, reviewed by Yan et al. [81], and how its biosynthesis switches between CS and CAS based on the availability of sulfur. Figure 2B shows L-Glutamic acid analogues that can interfere with glutamic acid action [2]. Since the trend in sulfur content on agriculture farms is decreasing [82], CS could be a key enzyme to improve cysteine in the future.

β-ODAP synthase (BOS), a crucial enzyme responsible for catalyzing the last stage of β-ODAP formation in grass pea, was isolated and characterized by Glodsmith et al. [23]. Additionally, Edwards et al. [24] employed a genomics approach to identify the CS in grass pea. These discoveries have opened new possibilities for utilizing gene editing techniques to decrease the β-ODAP content in grass pea. Both CAS and BOS can be targeted for genome editing to reduce the level of β-ODAP. Although L-DAPA is stable in vitro, it is a short-lived metabolic intermediate in grass pea and cannot be extracted from the grass pea [24]. This might be due to the faster reaction of β-ODAP synthase in converting the metabolite intermediate L-DAPA into β-ODAP. According to Goldsmith et al. [23], more than 99% of the L-DAPA is converted to β-ODAP, whilst fα-ODAP is less than 1%. They suggested that mutating the highly conserved Asp166 and His162 residues in β-ODAP synthase may result in an inactive form of this enzyme. Even though mutating β-ODAP synthase may help in studying L-DAPA intermediate and the nature of its stability in grass pea, this may not address the overall trends as its substrate may also have adverse effects.

## 5. Mechanism of β-ODAP Action and Toxicity

Since the discovery of β-ODAP, researchers have been investigating the potential mechanisms that could lead to toxicity. Ross et al. [83] examined the effects of β-ODAP on the central nervous system’s high-affinity transport for glutamate, gamma-aminobutyric acid (GABA), aspartate, glycine, choline, and glutamate decarboxylase (GAD), using rats as a model. Their findings indicated that, as the concentration of β-ODAP increased, aspartate transport in brain and spinal cord synaptosomes decreased, with glutamate transport dropping to 74% in the brain and 60% in the spinal cord compared to the control group. According to Krogsgaard-Larsen et al. [84], L-Glutamic acid (Glu) is the major excitatory amino acid neurotransmitter in the central nervous system (CNS) that activates at least three types of receptors. These are the N-Methyl-D-aspartic acid receptor (NMDA), α-amino-3-hydroxy-5-methyl-4-isoxazolepropionic acid receptor (AMPA), and kainic acid receptor (KAR) that mediate the neurotoxicity of several naturally occurring Glu analogs, including β-ODAP. β-ODAP was reported to act as an agonist of AMPA receptors, which resulted in the accumulation of intracellular calcium ions up to a toxic level [84]. Since the 1960s, efforts have been made to investigate the potential role of β-ODAP as a glutamate analogy and its interaction with glutamate receptors, and neuron signaling. However, an attempt to fully understand the molecular mechanism of neurolathyrism pathology at the cellular level is still lacking [1].

Though detailed in vivo mechanism of paralysis triggered by β-ODAP is still not fully understood, studies have used an animal cell model and animals to decipher its mechanism of action. β-ODAP acts as an excitatory amino acid in the same way as L-glutamic acid and AMPA receptors and triggers motor neuron degeneration by inducing excitotoxic cell death and increasing oxidative stress [40]. The toxin is known to disturb the mitochondrial respiration chain and inhibit the uptake of cystine, thereby affecting the cells’ abilities to cope with oxidative stress [40]. One of the mechanisms of glutamate excitotoxicity is Ca^2+^ overload-induced cell death. Ca^2+^ was proposed in [40] as a key player of β-ODAP-induced excitotoxicity and oxidative stress due to increased reactive oxygen species (ROS) that results in motor neuron degeneration paralysis. Accordingly, β-ODAP disturbs the cellular Ca^2+^ homeostatic machinery with increased Ca^2+^ loading in the endoplasmic reticulum (ER)-mitochondrial axis [40]. Motor neurons are very vulnerable to an increase in intracellular calcium due to the low content of Ca^2+^ binding proteins [85]. β-ODAP triggers Ca^2+^ accumulation and cell death in primary motor neurons through transient receptor potential channels and metabotropic glutamate receptors [86].

The experimental study conducted by Tan et al. [1] has widened our understanding of the molecular mechanism of β-ODAP toxicity using a cell model. Tan et al. showed that human glioma cell lines treated with β-ODAP resulted in decreased mitochondrial membrane potential by increasing Ca^2+^ in the cellular matrix and resulted in the overexpression of β1 integrin on the cytomembrane surface and resulted in oxidative stress due to the interruption of the electron transport chain in the mitochondria. Specifically, β-ODAP combines with AMPA receptors on the cell surface and abnormally activates the receptors and intracellular homeostasis through the inflow of Ca^2+^ [1]. Most β-ODAP-affected people in Ethiopia believe that milk consumption aggravates β-ODAP toxicity (personal communication), which may be due to the high amount of calcium in milk, but this needs further investigation. The *neurolathyrism* work conducted by Khandare et al. [87] has shown that goats fed milk supplemented with 0.17–0.96% of β-ODAP containing grass pea powder were affected by paralysis.

**Figure 3:**
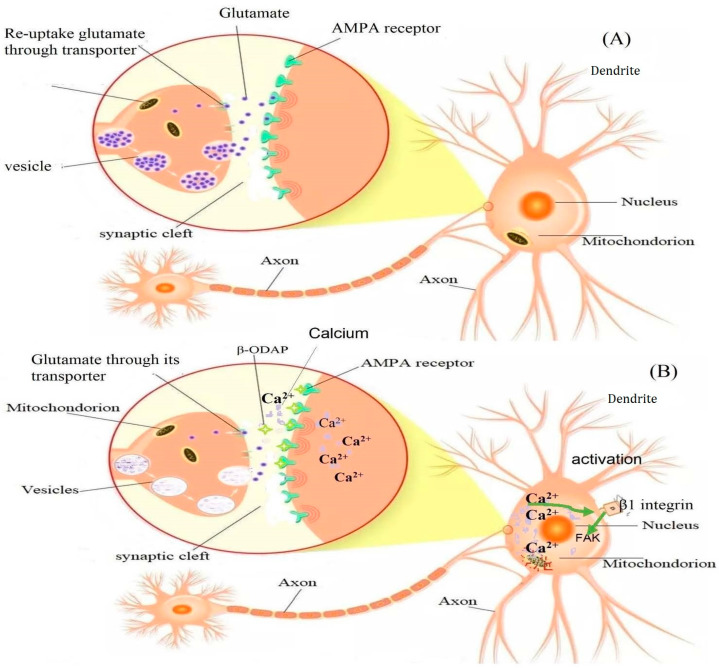
The mechanism of degeneration induced by β-ODAP proposed by [1], with modification of figure design. Figure (**A**) illustrates the removal of excitatory amino acid glutamate by glutamate transporter and its storage in a vesicle until needed. Figure (**B**) indicates the binding of β-ODAP to the AMPA receptor, which allows the influx of Ca^2+^ into the cell. The binding of β-ODAP and over-activating AMPA receptors triggers the signaling cascade of reactions with intracellular Ca^2+^ low. Meanwhile, the increased expression level of β1 integrin on the cell surface substantially induces the phosphorylation level of FAK and overexpression of paxillin. This causes massive aggregation of FA units on cell actin filaments, interferes with the assembly of cell microfilaments, and ultimately results in structural damage to the cells [1].

**Figure 4:**
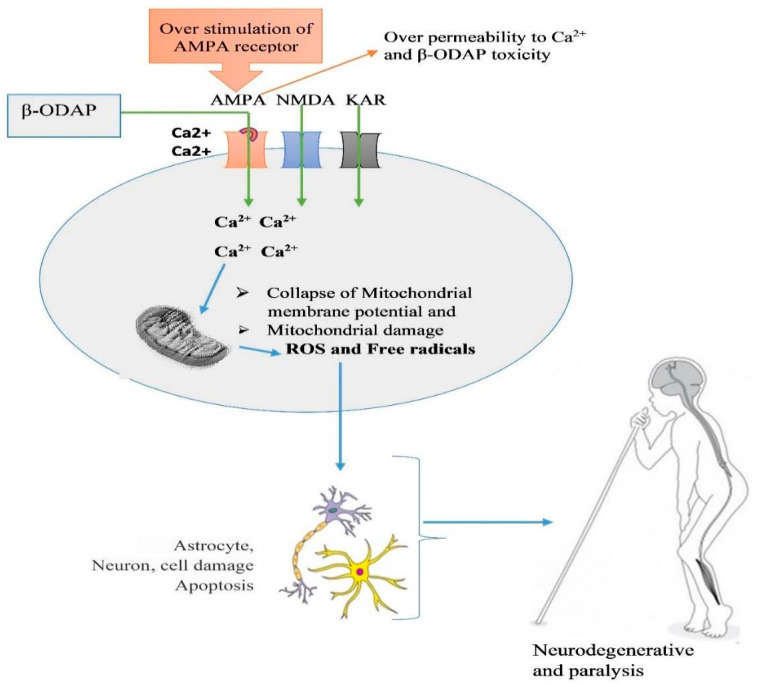
Illustration of β-ODAP toxicity and irreversible paralysis caused by extensive consumption of grass pea, as reported by a number of studies [80,91,92]. The model of lathyrism in humans was taken from (https://en.wikipedia.org/wiki/Konzo, accessed on 19 September 2024).

In normal physiological glutamate signaling (in the absence of β-ODAP), presynaptically released glutamate binds to surface receptors on postsynaptic neurons and activates them by inducing depolarization and an intracellular Ca^2+^ increase. Glutamate is then removed from the synaptic cleft by glutamate transporters (excitatory amino acid transporters; EAAT), located in the plasma membrane of both neurons and astrocytes. However, β-ODAP cannot be removed by glutamate transporters, and its binding allows more Ca^2+^ influx [88,89]. In astrocytes, glutamate is converted into glutamine–by-glutamine synthetase. Glutamine is released into the extracellular space, taken up by presynaptic neurons, and converted into glutamate by glutaminase. Glutamate is then stored in presynaptic vesicles, ready for the next glutamate–glutamine cycle [88,89]. β-ODAP competes with L-glutamic acid (Glu) to bind to the AMPA receptor. Furthermore, activation of AMPA receptors is reported to enhance β1 integrin expression and further increase the focal adhesion kinase (FAK) phosphorylation level [1]. On the other hand, β-ODAP toxicity was shown to be reduced by overexpression of Glutaredoxin 1 (Grx1), an important cytosolic thiol-disulfide oxidoreductase, which reduces glutathionylated proteins to protein thiols and helps to maintain the redox status of proteins during oxidative stress [90]. Figure 3 summarizes the possible mechanism of β-ODAP action [1] and Figure 4 summarizes overall toxicity from the cellular to organismal level.

## 6. Reducing β-ODAP Using Traditional Food Processing Strategies

Farmers usually use traditional food processing strategies to reduce toxic compounds in grass pea. Studies have shown that pre-processing by long soaking (>18 h) and fermentation significantly reduce the toxicant [93]. Using condiments such as onion and ginger in grass pea food preparation has been traditionally used to reduce its toxicity [94]. Furthermore, the traditional way of soaking grass pea seeds in various media has been shown to reduce the contents of β-ODAP to a varying and significant extent [93]. For instance, the loss of β-ODAP was significantly higher when soaked in boiling water (65–70%), rather than in cold water [93]. Some chemicals, like polyphenols, have been suggested to lower the toxicity of grass pea, and they can be applied in a feasible and effective manner [93]. Meanwhile, Tadele et al. [95] suggested that cooking grass pea for 20 min and roasting is important in reducing and eliminating anti-nutritional factors like β-ODAP. According to a study by Akalu et al. [96], roasting and autoclaving grass pea reduced the content of β-ODAP by up to 30% and 50%, respectively, while Buta et al. [97] reported that soaking grass pea seeds under high hydrostatic pressure resulted in a 36–71% reduction in β-ODAP. However, a more feasible, sustainable, and scalable approach is needed for farmers to reduce or eliminate toxic compounds. Hence, the production of toxic-free grass pea is of paramount importance to address all safety concerns related to this important stress-resilient crop without compromising its nutritional value.

## 7. Enhancing Sulfur-Containing Amino Acids (SCA) through Genetic Modification of Grass Pea

Despite its high protein content, grass pea is limited by its low levels of sulfur-containing amino acids (SCA), such as methionine and cysteine. As mentioned earlier, under low sulfur conditions, the intermediate BIA is predominantly synthesized from isoxazoline-5-one and O-acetyl-serine, with the help of cysteine synthase. To address this limitation, genetic engineering of grass pea can be explored by introducing genes that can enhance SCA levels. This approach may lead to the development of biofortified and toxin-free grass pea. A reproducible transformation protocol has been developed for Indian grass pea accession using epicotyl segment co-cultivation with Agrobacterium strains EHA 105 and LBA 4404 [98]. The biolistic transformation of grass pea somatic embryos was also reported as a potential tool for grass pea transformation [99]. The transient genetic transformation of two grass pea varieties has also been reported in Ethiopia [100]. Therefore, by introducing candidate genes that can boost SCA through heterologous expression, the amino acid content of grass pea can be improved.

Various genes, including Brazil nut 2S protein (BN2S) [101], sunflower 2S albumin (SFA8) [102], and δ-zein protein [103,104], have been identified and reported as potential candidates for boosting SCA. For instance, the introduction of a methionine-rich 10-kDa δ-zein gene into transgenic maize resulted in an increase of 57.6% in methionine content [105]. Similarly, the engineering of the 15-kDa δ-zein gene in soybean led to a 12–20% increase in methionine and a 15–35% increase in cysteine [106]. Another promising candidate gene is the 11 kDa δ-zein protein, which contains 32 methionine and 7 cysteine residues, accounting for 25% of the total amino acids in the protein [107]. Similarly, the introduction of the β-zein gene into soybean resulted in an increase of up to 15% in methionine content [108].

The utilization of enzymatic genes such as ATP sulfurylase, which aids in the conversion of sulfate to adenosine 5′-phosphosulfate (APS), may play a role in regulating sulfur metabolism and improving SCA. The overexpression of ATP sulfurylase resulted in significant improvements, with a 37–52% increase in protein-bound cysteine content and a 15–19% increase in methionine content [109]. In a study conducted by Kin et al. [110], a cytosolic isoform of O-acetylserine sulfhydrylase (OASS) was introduced into transgenic soybeans, leading to a noteworthy 22–32% elevation in free cysteine levels in the soybeans. Furthermore, Avraham et al. [111] conducted another study where they introduced Arabidopsis cystathionine γ-synthase (AtCGS) into alfalfa plants, resulting in a remarkable 32-fold increase in methionine and a 2.6-fold increase in free cysteine, indicating a potential to increase the SCA content in grass pea, thereby generating biofortified grass pea lines.

## 8. The Potential of the Genome Editing Approach for Antinutritional Factors in Crops

Genome editing in plants has opened new opportunities to incorporate novel traits beneficial for human health and the environment by eliminating antinutritional factors. Genome editing encompasses techniques such as oligonucleotide-directed mutagenesis (ODM) and site-directed nucleases (SDNs), like zinc finger nucleases (ZFNs), transcription activator-like effector nucleases (TALENs), and mega-nucleases and clustered regularly interspaced short palindromic repeats/CRISPR-associated (CRISPR/Cas) techniques [112]. Unlike the random traditional mutagenesis approach, site-directed nucleases (SDN) are playing a pivotal role in precise genome edition as they are faster and more targeted [113]. Depending on the nature of the edit, genome editing with the SDN process can be categorized under SDN1, SDN2, or SDN3 [114]. The SDN1 involves the unguided repair of a targeted double-strand break (DSB) with small indels, and joins ends in a non-homologous manner. The repair of this break can lead to a mutation causing gene silencing through loss of function without the addition of foreign DNA but due to a shift in the reading frame. SDN2 involves a template-guided repair of a targeted DSB, typically short single-stranded DNA, while SDN3 involves a template-guided repair of a targeted DSB, typically with double-stranded DNA with longer genetic elements [115]. Studies indicate that SDN1 and SDN2 do not require foreign DNA for genome editing, whilst SDN3 requires foreign DNA segments [114,115,116].

CRISPR-associated Cas9 endonuclease is becoming available in genome editing technology that enables precise modification in the genome. CRISPR/Cas9 has advantages over other gene editing because it is relatively less costly, more precise, and the operation is easier and more efficient, allowing researchers to induce targeted knockouts [116,117]. In recent years, CRISPR/Cas9 has been used successfully for genome editing in more than 20 crop plants to alter various characteristics, such as increasing yield, promoting growth, and removing unwanted anti-nutritional factors [118,119,120]. This method can be used to create knockouts of non-important enzymatic functions, which in many cases has been difficult to achieve with the traditional genetic engineering approach. In sweet potato, CRISPR/Cas9 has been used to knockout *SBEII* and increase the amylose percentage [121,122]. High-amylose and resistant starch wheat was also generated through the suppression of *TaSBEIIa* in a modern winter and spring wheat using CRISPR/Cas9 [123,124]. Very recently, cytochrome P450 genes CYP79D1 and CYP79D2, which accumulate toxic cyanogen in cassava, were successfully edited using CRISPR-Cas9-mediated knockout [125].

Phytic acid (PA) is an antinutrient in cereal grains that inhibits the absorption of micronutrients like iron and zinc in humans, leading to malnutrition. While PA is a significant source of phosphorus in plants, it is considered detrimental for monogastric animals, including humans, due to its negative effects on essential mineral uptake [126]. Through the targeting of the enzyme *Inositol-pentakisphosphate 1-kinase* (IPK1), which participates in the catalysis of the final step of PA biosynthesis in soybean (*Glycine max* L.), using two guide RNAs directed at the second and third exons of GmIPK1, the PA content was reduced by 25% without adverse effects on plant growth or seed development [126]. Likewise, CRISPR-Cas9 was applied to tetraploid *Brassica napus* to knock out three functional paralogs of *BnITPK*, which resulted in a higher reduction of PA (~35%) in triple mutants [127].

Despite being an essential amino acid, crops like wheat contain elevated levels of free asparagine in their grains, which can form acrylamide during high-temperature cooking processes, a known carcinogenic compound [127]. By introducing four gRNAs targeting the wheat asparagine synthetase gene (*TaASN2*) into wheat embryos using CRISPR/Cas9, Raffan et al. [128] reported a reduction of free asparagine by approximately 90%. Asparagine is also a precursor of β-ODAP biosynthesis.

In cotton (*Gossypium hirsutum)*, gossypol is a polyphenol that accumulates in the seeds. Gossypol can lead to acute poisoning when consumed by various animals, such as broiler chicks, pigs, dogs, sheep, and goats, due to its antispermatogenic activity, raising health concerns [129]. The utilization of CRISPR-Cas9 has been shown to successfully knock out dirigent protein genes GhDIR5 and GhDIR6, reducing cotton seed toxicity without affecting other phytoalexins [130]. Additionally, CRISPR-Cas9 was employed to significantly decrease nicotine levels in tobacco by 99.6%, aiming to develop nicotine-free tobacco by targeting the flavoproteins of the *berberine bridge enzyme-like* (*BBL*) gene responsible for the final oxidation step in nicotine biosynthesis [131]. Furthermore, CRISPR-Cas9 was effectively used in the medicinal plant comfrey (*Symphytum officinale*) to eliminate toxic pyrrolizidine alkaloids by introducing detrimental mutations into the gene encoding *homospermidine synthase* (*HSS*) [132]. In legumes, the CRISPR/Cas9-mediated edition of the GmFATB1 gene significantly reduced the amount of saturated fat in soybean [133] and the level of trypsin inhibitor by targeting KTI1 and KTI3 genes [134] in soybean. Hence, CRISPR/Cas9 can be applied to other crops, such as grass, to reduce or eliminate anti-nutrient content.

## 9. The Potential of CRISPR/Cas9 to Target β-ODAP Biosynthesis in Grass Pea

The availability of a draft genome sequence of grass pea [24,117,135] can now allow researchers to develop a novel strategy to reduce β-ODAP toxicity. It is becoming clearer that the enzymes involved in the biosynthesis of β-ODAP are potential candidates for CRISPR/Cas9-based genome editing. The β-ODAP synthase (BOS) gene (AC: MT457411) is a 1320 bp mRNA encoding 439 amino acids. Analysis of the conserved domains of BOS has shown that amino acids from positions 3–434 have a role in transferase activity (E value = 5.11 × 10^−75^). Hence, a single nucleotide deletion in this gene can lead to frameshift, blocking the synthesis of β-ODAP from L-DAPA and oxalyl-CoA following the first strategy. We propose four potential strategies to decrease or eliminate β-ODAP biosynthesis in grass pea. The first strategy is to target the BOS gene only. The second strategy is to target the CAS gene only. The third strategy is to simultaneously target both BOS and CAS genes. The fourth strategy is replacing specific amino acids in either BOS or CAS, either separately or together, to partially reduce β-ODAP biosynthesis. The latter strategy is suggested if the complete or partial knockout of β-ODAP biosynthesis has undesirable phenotypes, such as sensitivity to abiotic stresses like drought. In the first strategy (targeting BOS), further accumulation of the precursor L-DAPA must also be considered to monitor the level of toxic intermediate. The in vitro toxicological effects of isoxazoline amino acids of *Lathyrus sativus* were investigated by Riepe et al. [136]. Their finding suggested that 2000 µM of BIA resulted in cell damage and neurodegeneration in a mouse cortical similar to 50 µM β-ODAP in a concentration-dependent manner. This is a good indication that the intermediate BIA is 40 fold less toxic when compared to β-ODAP and may not be a major concern. However, further study is needed to determine the level of toxin precursor accumulation after gene knockout. The recent pre-print article has reported an attempt to reduce β-ODAP by targeting the *BAHD-AT3* gene [137]. Furthermore, hairy root transformation was used for grass pea transformation to edit Oxalyl-CoA Synthetase [138].

**Figure 5:**
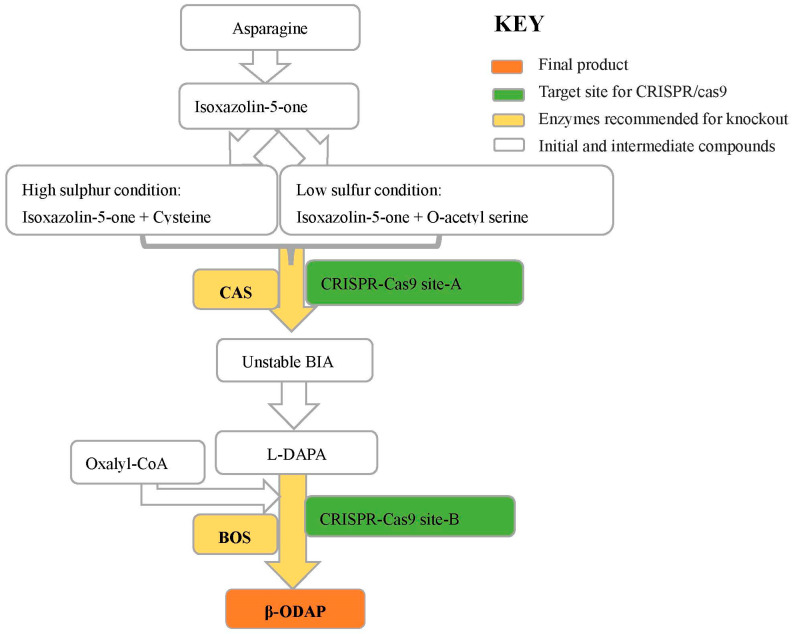
Potential target for CRISPR/cas9 for β-ODAP reduction in grass pea. Abbreviations: L-DAPA represent L-2,3-diaminopropionic acid; BIA is β-(isoxazolin-5-on-2-yl) alanine); BOS is β-ODAP synthase; and CAS is β-cyanoalanine synthase. CAS and BOS are potential target genes that can be knocked out either simultaneously or separately.

If L-DAPA and BIA show significant toxicity, similar to the intermediate precursors, β-cyanoalanine synthase (CAS) can be another target for gene editing. CAS has been shown to synthesize the intermediate BIA from isoxazoline-5-one and cysteine catalyzed [46] under high-sulfur conditions. In grass pea, the CAS gene (AC: KJ563188) is a 1146 bp complete mRNA that encodes 381 amino acids. The cloning of the CAS gene from *L. sativus* (GenBank: KJ563188), which has 99.74% identity with KJ563188 at the protein level, was reported by Xu et al. [80]. Figure 5 depicts the possible gene targets for CRISPR/Cas9.

**Figure 6:**
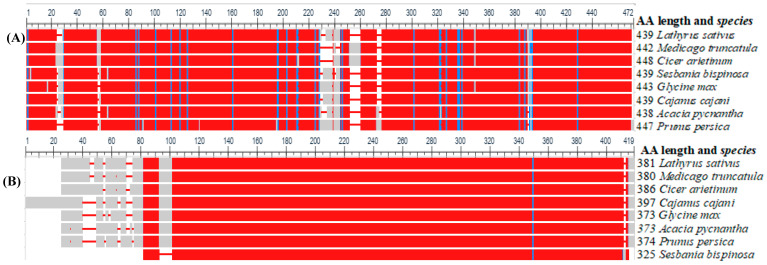
Similarity of grass pea (*Lathyrus sativus*) β-ODAP synthase enzymes (**A**) and β-cyanoalanine synthase (**B**) with other plants. Solid red color indicates conserved amino acids in all the eight species, the grey and white columns represent indels, and the blue line represents the substitution of aligned sequences. Amino acid length and names of all the species are listed on the right side of the figure.

To further understand the similarity of BOS and CAS enzymes in seven legumes and a rosaceae family, we computed multiple sequence alignment using COBALT (constraint-based alignment tool for multiple protein sequences), which computes multiple protein sequence alignments using conserved domain and local sequence similarity information [139]. Figure 6 shows the similarity of grass pea BOS and CAS with homologs in other plant species.

**Figure 7:**
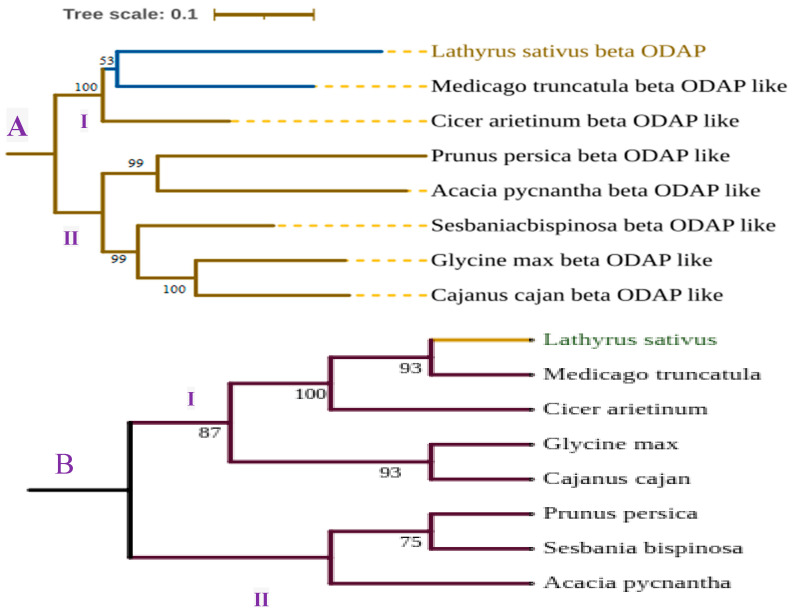
Genetic relatedness of β-ODAP ((**A**) scaled tree) and CAS enzyme ((**B**) unscaled tree) as compared to legume models and other plants. With both enzymes, *L. sativus* and *M. truncatula* have shown a close relationship, though there are some amino acid differences in conserved areas.

As indicated in Figure 6, CAS has shown strong similarity among aligned proteins. The *Lathyrus sativus* CAS and a model *Medicago truncatula* showed 95% similarity, followed by CAS of *Glycine max* and *Cajanus cajani* (94%). The lowest similarity was observed between the *Prunus persica* and *Sesbania bispinosa* CAS genes. Furthermore, remarkable variability was shown in the conserved domain of BOS among selected plants. Using the BOS gene, *L. sativum* and *M. truncatula* have a similarity of 62.9% at the protein level, indicating the existence of significant variability from model legumes. The BOS of *C. cajani* and *G. max* has shown a 73.5% similarity, followed by *M*. *truncatula* and *Cicer arietinum* (72.8%). The variability of β-ODAP in grass pea might have resulted in enhanced enzyme activity to convert the intermediate L-DAPA into more toxic β-ODAP selectively in grass pea, which needs further empirical study. The scaled and unscaled phylogenetic tree (Figure 7) depicts the genetic relatedness of those two enzymes in different crop plants, including model legume. The trees were constructed using MEGA 11 [140] and edited by ITOLv5 (Interactive tree of life) [141]. Overall, the β-ODAP of *L. sativus* and the model legume *M. truncatulaare* are strongly related to each other compared with other species used for tree construction.

## 10. Conclusions and Prospects

Grass pea is a stress-tolerant, nutritious crop that has a high level of the antinutritional factor β-ODAP. This review summaries the benefits of grass pea in adapting to abiotic stresses affected by ecological zones as the future crop of choice in the ever-changing global environment. As grass pea is poor in sulfur-containing amino acids (SCA), the ideal genes capable of boosting SCA were also highlighted. The biosynthesis of toxic compounds in grass pea, the molecular mechanisms of their toxicity, and the potential of CRISPR/Cas9 technology in reducing grass pea toxicity has been discussed. To fully benefit from grass pea, toxic compounds need to be reduced or eliminated by targeting the key enzymes responsible for β-ODAP biosynthesis via a CRISPR/Cas9 genome editing approach. The knocking out of BOS and/or CAS enzymes can help in reducing β-ODAP biosynthesis. Given its high-yielding potential and stress tolerance, grass pea production needs to be expanded to increase food security in the face of global change. Therefore, implementing modern biotechnologies like CRISPR/Cas9 is vital for removing antinutritional factors from this legume. Future research should focus on the genes and strategies discussed in this article to produce β-ODAP-free grass pea varieties.

## Data Availability

All data used in this manuscript are included in the main manuscript.

## References

[1] Tan R.-Y., Xing G.-Y., Zhou G.-M., Li F.-M., Hu W.-T., Lambein F., Xiong J.-L., Zhang S.-X., Kong H.-Y., Zhu H. (2017). Plant toxin β-ODAP activates integrin β1 and focal adhesion: A critical pathway to cause neurolathyrism. Sci. Rep..

[2] Lambein F., Travella S., Kuo Y.-H., Van Montagu M., Heijde M. (2019). Grass pea (*Lathyrus sativus* L.): Orphan crop, nutraceutical or just plain food?. Planta.

[3] Gonçalves L., Rubiales D., Bronze M.R., Vaz Patto M.C. (2022). Grass Pea (*Lathyrus sativus* L.)—A Sustainable and Resilient Answer to Climate Challenges. Agronomy.

[4] Smartt J. (1990). Pulses of the classical world. Grain Legumes: Evaluation and Genetic Resources.

[5] Abdipour M., Vaezi B., Khademi K., Ghasemi S. (2019). An optimized artificial intelligence approach and sensitivity analysis for predicting the biological yield of grass pea (*Lathyrus sativus* L.). Arch. Agron. Soil Sci..

[6] Sammour R.H. (2014). Genetic diversity in *Lathyrus sativus* L. germplasm. Res. Rev. BioSci..

[7] Gupta P., Udupa S.M., Gupta D.S., Kumar J., Kumar S. (2018). Population structure analysis and determination of neurotoxin content in a set of grass pea (*Lathyrus sativus* L.) accessions of Bangladesh origin. Crop J..

[8] Verma S.C., Ohri D. (1979). Chromosome and nuclear phenotype in the legume *Lathyrus sativus* L.. Cytologia.

[9] Hanbury C., White C., Mullan B., Siddique K. (2000). A review of the potential of *Lathyrus sativus* L. and *L. cicera* L. grain for use as animal feed. Anim. Feed. Sci. Technol..

[10] Vaz Patto M.C., Skiba B., Pang E.C.K., Ochatt S.J., Lambein F., Rubiales D. (2006). Lathyrus improvement for resistance against biotic and abiotic stresses: From classical breeding to marker assisted selection. Euphytica.

[11] Almeida N., Rubiales D., Vaz Patto M., De Ron A. (2015). Grass Pea. Grain Legumes. Handbook of Plant Breeding.

[12] Shiferaw E., Pè M.E., Porceddu E., Ponnaiah M. (2011). Exploring the genetic diversity of Ethiopian grass pea (*Lathyrus sativus* L.) using EST-SSR markers. Mol. Breed..

[13] Dadi L., Teklewold H., Aw-Hassan A., Moneim A.A., Bejiga G. (2003). The Socioeconomic Factors Affecting Grass Pea Consumption and Influence of Lathyrism in Ethiopia.

[14] Gabrekiristos E., Wondimu M. (2022). Emerging and Reemerging Diseases of Common Bean (*Phaseolus vulgaris* L.) in major production areas: In the case of Ethiopia. J. Plant Pathol. Microbiol..

[15] Sarkar A., Emmrich P.M.F., Sarker A., Zong X., Martin C., Wang T.L., Kole C. (2019). Grass pea: Remodelling an ancient insurance crop for climate resilience. Genomic Designing of Climate-Smart Pulse Crops.

[16] Fikre A., Van Moorhem M., Ahmed S., Lambein F., Gheysen G. (2011). Studies on neurolathyrism in Ethiopia: Dietary habits, perception of risks and prevention. Food Chem. Toxicol..

[17] Arslan M., Basak M., Aksu E., Uzun B., Yol E. (2020). Genotyping of Low β-ODAP Grass Pea (*Lathyrus sativus* L.) Germplasm with EST-SSR Markers. Braz. Arch. Biol. Technol..

[18] Spencer P.S., Roy D.N., Ludolph A., Hugon J., Dwivedi M.P., Schaumburg H.H. (1986). Lathyrism: Evidence for role of the neuroexcitatory amino acid BOAA. Lancet.

[19] Das A., Parihar A.K., Barpete S., Kumar S., Gupta S. (2021). Current Perspectives on Reducing the β-ODAP Content and Improving Potential Agronomic Traits in Grass Pea (*Lathyrus sativus* L.). Front. Plant Sci..

[20] Banerjee J., Das A., Parihar A.K., Sharma R., Pramanik K., Barpete S., Kole C. (2022). Genomic Designing Towards Development of Abiotic Stress Tolerant Grass Pea for Food and Nutritional Security. Genomic Designing for Abiotic Stress Resistant Pulse Crops.

[21] Rizvi A.H., Sarker A., Dogra A. (2016). Enhancing grasspea (*Lathyrus sativus* L.) production in problematic soils of South Asia for nutritional security. Indian J. Genet. Plant Breed..

[22] Rao S.L., Adiga P.R., Sarma P.S. (1964). The Isolation and Characterization of β-N-Oxalyl-L-α,β-Diaminopropionic Acid: A Neurotoxin from the Seeds of *Lathyrus sativus*. Biochemistry.

[23] Goldsmith M., Barad S., Knafo M., Savidor A., Ben-Dor S., Brandis A., Mehlman T., Peleg Y., Albeck S., Dym O. (2022). Identification and characterization of the key enzyme in the biosynthesis of the neurotoxin β-ODAP in grass pea. J. Biol. Chem..

[24] Edwards A., Njaci I., Sarkar A., Jiang Z., Kaithakottil G.G., Moore C., Cheema J., Stevenson C.E.M., Rejzek M., Novák P. (2023). Genomics and biochemical analyses reveal a metabolon key to β-L-ODAP biosynthesis in *Lathyrus sativus*. Nat. Commun..

[25] Rathi D., Chakraborty S., Chakraborty N. (2021). Grasspea, a critical recruit among neglected and underutilized legumes, for tapping genomic resources. Curr. Plant Biol..

[26] Urga K., Fufa H., Biratu E., Husain A. (2005). Evaluation of *Lathyrus sativus* cultivated in Ethiopia for proximate composition, mineral and b-ODAP and antinutritional components. Afr. J. Food Agric. Nutr. Dev..

[27] Chandna M., Matta N.K. (1994). Studies on Changing Protein Levels in Developing and Germinating Seeds of *Lathyrus sativus* L.. J. Plant Biochem. Biotechnol..

[28] Grela E.R., Rybiński W., Klebaniuk R., Matras J. (2010). Morphological characteristics of some accessions of grass pea (*Lathyrus sativus* L.) grown in Europe and nutritional traits of their seeds. Genet. Resour. Crop Evol..

[29] Arslan M. (2017). Diversity for vitamin and amino acid content in grass pea (*Lathyrus sativus* L.). Legume Res..

[30] Rao S.L.N., Ramachandran L.K., Adiga P.R. (1963). The isolation and characterization of L-homoarginine from seeds of *Lathyrus sativus*. Biochemistry.

[31] Rao S. (2011). A look at the brighter facets of β-N-oxalyl-L-α,β-diaminopropionic acid, homoarginine and the grass pea. Food Chem. Toxicol..

[32] Tsikas D., Wu G. (2015). Homoarginine, arginine, and relatives: Analysis, metabolism, transport, physiology, and pathology. Amino Acids.

[33] Rodionov R.N., Begmatov H., Jarzebska N., Patel K., Mills M.T., Ghani Z., Khakshour D., Tamboli P., Patel M.N., Abdalla M. (2019). Homoarginine Supplementation Prevents Left Ventricular Dilatation and Preserves Systolic Function in a Model of Coronary Artery Disease. J. Am. Heart Assoc..

[34] Jammulamadaka N., Burgula S., Medisetty R., Ilavazhagan G., Rao S.L.N., Singh S.S. (2011). β-*N*-oxalyl-l-α,β-diaminopropionic acid regulates mitogen-activated protein kinase signaling by down-regulation of phosphatidylethanolamine-binding protein 1. J. Neurochem..

[35] May M., Kayacelebi A.A., Batkai S., Jordan J., Tsikas D., Engeli S. (2015). Plasma and tissue homoarginine concentrations in healthy and obese humans. Amino Acids.

[36] Tsikas D., Bollenbach A., Hanff E., Kayacelebi A.A. (2018). Asymmetric dimethylarginine (ADMA), symmetric dimethylarginine (SDMA) and homoarginine (hArg): The ADMA, SDMA and hArg paradoxes. Cardiovasc. Diabetol..

[37] Llorent-Martínez E.J., Zengin G., Fernández-de Córdova M.L., Bender O., Atalay A., Ceylan R., Mollica A., Mocan A., Uysal S., Guler G.O. (2017). Traditionally used Lathyrus species: Phytochemical composition, antioxidant activity, enzyme inhibitory properties, cytotoxic effects, and in silico studies of *L. czeczottianus* and *L. nissolia*. Front. Pharmacol..

[38] Striefler M., Cohn D., Hirano A., Schujman E. (1977). The central nervous system in a case of neurolathyrism. Neurology.

[39] Haimanot H., Kidane Y., Wuhib E., Kalissa A., Alemu T., Zein Z., Spencer P. (1990). Lathyrism in rural northwestern Ethiopia: A highly prevalent neurotoxic disorder. Int. J. Epidemiol..

[40] Van Moorhem M., Lambein F., Leybaert L. (2011). Unraveling the mechanism of β-N-oxalyl-α,β-diaminopropionic acid (β-ODAP) induced excitotoxicity and oxidative stress, relevance for neurolathyrism prevention. Food Chem. Toxicol..

[41] Getahun H., Mekonnen A., TekleHaimanot R., Lambein F. (1999). Epidemic of neurolathyrism in Ethiopia. Lancet.

[42] Woldeamanuel Y.W., Hassan A., Zenebe G. (2011). Neurolathyrism: Two Ethiopian case reports and review of the literature. J. Neurol..

[43] Giménez-Roldán S., Spencer P.S. (2016). Azañón’s disease. A 19th century epidemic of neurolathyrism in Spain. Rev. Neurol..

[44] Giménez-Roldán S., Palmer V.S., Spencer P.S. (2023). Lathyrism in Spain: Lessons from 68 publications following the 1936–39 Civil War. J. Hist. Neurosci..

[45] Girma A., Tefera B., Dadi L. (2011). Grass Pea and Neurolathyrism: Farmers’ perception on its consumption and protective measure in North Shewa, Ethiopia. Food Chem. Toxicol..

[46] Hoque H., Jamali S., Akther J., Prodhan S.H. (2012). Computational analysis of milk sources from different domestic animals as supplementary food source to protect Lathyrism. Int. J. Biosci..

[47] Girma D., Korbu L. (2012). Genetic improvement of grass pea (*Lathyrus sativus*) in Ethiopia: An unfulfilled promise. Plant Breed..

[48] Bell E., O’Donovan J.P. (1966). The isolation of α- and γ-oxalyl derivatives of α,γ-diaminobutyric acid from seeds of Lathyrus latifolius, and the detection of the α-oxalyl isomer of the neurotoxin α-amino-β-oxalylaminopropionic acid which occurs together with the neurotoxin in this and other species. Phytochemistry.

[49] Vaz Patto M.C., Rubiales D. (2014). Lathyrus diversity: Available resources with relevance to crop improvement—*L. sativus* and *L. cicera* as case studies. Ann. Bot..

[50] Dixit G.P., Parihar A.K., Bohra A., Singh N.P. (2016). Achievements and prospects of grass pea (*Lathyrus sativus* L.) improvement for sustainable food production. Crop J..

[51] Song Y., Wang L., Liu F., Jiao C., Nan H., Shen X., Chen H., Li Y., Lei B., Jiang J. (2021). β-Cyanoalanine Synthase Regulates the Accumulation of β-ODAP via Interaction with Serine Acetyltransferase in *Lathyrus sativus*. J. Agric. Food Chem..

[52] Kusama-Eguchi K., Yoshino N., Minoura A., Watanabe K., Kusama T., Lambein F., Ikegami F. (2011). Sulfur amino acids deficiency caused by grass pea diet plays an important role in the toxicity of L-β-ODAP by increasing the oxidative stress: Studies on a motor neuron cell line. Food Chem. Toxicol..

[53] Kumar S., Bejiga G., Ahmed S., Nakkoul H., Sarker A. (2011). Genetic improvement of grass pea for low neurotoxin (β-ODAP) content. Food Chem. Toxicol..

[54] McCutchan J.S. (2003). A brief history of grasspea and its use in crop improvement. Lathyrus Lathyrism Newsl..

[55] Rahman M., Ali M., Alam F., Banu M., Faruk M., Bhuiyan M. (2017). Biocontrol of foot and root rot disease of grasspea (*Lathyrus sativus*) by dual inoculation with rhizobium and arbuscular mycorrhiza. Bangladesh J. Microbiol..

[56] Campbell C.G., Briggs C.J. (1987). Registration of low neurotoxin content *Lathyrus* germplasm LS 8246. Crop. Sci..

[57] Yang H.M., Zhang X.Y. (2005). Considerations on the reintroduction of grass pea in China. Lathyrus Lathyrism Newsl..

[58] Tadesse W., Bekele E. (2003). Variation and association of morphological and biochemical characters in grass pea (*Lathyrus sativus* L.). Euphytica.

[59] Santha I.M., Mehta S.L. (2001). Development of low ODAP somaclones of *Lathyrus sativus*. Lathyrus Lathyrism Newsl..

[60] Yadav C.R. (1996). Genetic evaluation and varietal improvement of grasspea in Nepal. Lathyrus Genetic Resources in Asia: Proceedings of a Regional Workshop.

[61] Granati E., Bisignano V., Chiaretti D., Crinò P., Polignano G.B. (2003). Characterization of Italian and exotic *Lathyrus* germplasm for quality traits. Genet. Resour. Crop Evol..

[62] Krause D., Krause I. (2003). New green manuring *Lathyrus sativus* variety AC Greenfix available in USA. Lathyrus Lathyrism Newsl..

[63] Mera M., Tay J., France A., Montenegro A., Espinoza N., Gaete N. (2003). Luanco-INIA, a large-seeded cultivar of *Lathyrus sativus* released in Chile. Lathyrus Lathyrism Newsl..

[64] Sen Gupta D., Barpete S., Kumar J., Kumar S., Gupta D.S., Gupa S., Kumar J. (2021). Breeding for better grain quality in lathyrus. Breeding for Enhanced Nutrition and Bio-Active Compounds in Food Legumes.

[65] Malek M.A., Sarwar C.D.M., Sarker A., Hassan M.S., Arora R.K., Mathur P.N., Riley K.W., Adham Y. (1996). Status of grass pea research and future strategy in Bangladesh. Lathyrus Genetic Resources in Asia.

[66] Kumar S., Gupta P., Barpete S., Choukri H., Maalouf F., Sarkar A. (2021). Grass pea. The Beans and the Peas.

[67] ICARDA (2007). ICARDA Annual Report.

[68] Mehta S.L., Santha I.M., Kirti P.B. (2008). Somaclonal variation and genetic transformation in *Lathyrus sativus*. Handbook of New Technologies for Genetic Improvement of Legumes.

[69] Campbell C.G., Mehra R.B., Agrawal S.K., Chen Y.Z., El Moneim A.M.A., Khawaja H.I.T., Yadov C.R., Tay J.U., Araya W.A. (1993). Current status and future strategy in breeding grasspea (*Lathyrus sativus*). Euphytica.

[70] MoARD (2008). Animal and Plant Health Regulatory Directorate.

[71] Hussain M., Chowdhury B., Hoque R., Lambein F., Tekle Haimanot R., Lambein F. (1997). Effect of water stress, salinity, interaction of cations, stage of maturity of seeds and storage devices on the ODAP content of *Lathyrus sativus*. Lathyrus and Lathyrism a Decade of Progress.

[72] Xing G., Cui K., Li J., Wang Y., Li Z. (2001). Water stress and accumulation of β-N-oxalyl-L-α,β-diaminopropionic acid in grass pea (*Lathyrus sativus*). J. Agric. Food Chem..

[73] Jiao C.J., Xu Q.L., Wang C.Y., Li Z.X., Wang Y.F. (2006). Accumulation pattern of toxin β-ODAP during lifespan and effect of nutrient elements on β-ODAP content in *Lathyrus sativus* seedlings. J. Agric. Sci..

[74] Haque R.M., Kuo Y.-H., Lambein F., Hussain M. (2011). Effect of environmental factors on the biosynthesis of the neuro-excitatory amino acid β-ODAP (β-N-oxalyl-l-α,β-diaminopropionic acid) in callus tissue of *Lathyrus sativus*. Food Chem. Toxicol..

[75] Tokarz B., Wójtowicz T., Makowski W., Jędrzejczyk R.J., Tokarz K.M. (2020). What is the Difference between the Response of Grass Pea (*Lathyrus sativus* L.) to Salinity and Drought Stress?—A Physiological Study. Agronomy.

[76] Malathi K., Padmanaban G., Rao S.L., Sarma P.S. (1967). Studies on the biosynthesis of β-N-oxalyl-L-α, β-diaminopropionic acid, the *Lathyrus sativus* neurotoxin. Biochim. Biophys. Acta.

[77] Malathi K., Padmanaban G., Sarma P.S. (1970). Biosynthesis of β-N-Oxalyl-L-α,β-Diaminopropionic acid, *Lathyrus sativus* neurotoxin. Phytochemistry.

[78] Ikegami F., Ongena G., Sakai R., Itagaki S., Kobori M., Ishikawa T., Kuo Y.-H., Lambein F., Murakoshi I. (1993). Biosynthesis of β-(isoxazolin-5-on-2-yl)-l-alanine by cysteine synthase in *Lathyrus sativus*. Phytochemistry.

[79] Kuo J.M., Raushel F.M. (1994). Identification of the histidine ligands to the binuclear metal center of phosphotriesterase by site-directed mutagenesis. Biochemistry.

[80] Xu Q., Liu F., Chen P., Jez J.M., Krishnan H.B. (2017). β-*N*-Oxalyl-l-α,β-diaminopropionic acid (β-ODAP) content in *Lathyrus sativus*: The integration of nitrogen and sulfur metabolism through β-cyanoalanine synthase. Int. J. Mol. Sci..

[81] Yan Z.-Y., Spencer P.S., Li Z.-X., Liang Y.-M., Wang Y.-F., Wang C.-Y., Li F.-M. (2006). *Lathyrus sativus* (grass pea) and its neurotoxin ODAP. Phytochemistry.

[82] Feinberg A., Stenke A., Peter T., Hinckley E.-L.S., Driscoll C.T., Winkel L.H.E. (2021). Reductions in the deposition of sulfur and selenium to agricultural soils pose risk of future nutrient deficiencies. Commun. Earth Environ..

[83] Ross S.M., Roy D.N., Spencer P.S. (1985). β-N-Oxalylamino-L-alanine: Action on high-affinity transport of neurotransmitters in rat brain and spinal cord synaptosomes. J. Neurochem..

[84] Krogsgaard-Larsen P., Hansen J.J. (1992). Naturally-occurring excitatory amino acids as neurotoxins and leads in drug design. Toxicol. Lett..

[85] Grosskreutz J., Van Den Bosch L., Keller B. (2010). Calcium dysregulation in amyotrophic lateral sclerosis. Cell Calcium.

[86] Kusama-Eguchi K., Miyano T., Yamamoto M., Suda A., Ito Y., Ishige K., Ishii M., Ogawa Y., Watanabe K., Ikegami F. (2014). New insights into the mechanism of neurolathyrism: L-β-ODAP triggers [Ca^2+^]_i_ accumulation and cell death in primary motor neurons through transient receptor potential channels and metabotropic glutamate receptors. Food Chem. Toxicol..

[87] Khandare A.L., Kalakumar B., Validandi V., Ssyh Q., Harishankar N., Singh S.S., Kodali V. (2020). Neurolathyrism in goat (*Capra hircus*) kid: Model development. Res. Vet. Sci..

[88] Doble A. (1999). The role of excitotoxicity in neurodegenerative disease: Implications for therapy. Pharmacol. Ther..

[89] Hertz L. (2006). Glutamate, a neurotransmitter–and so much more A synopsis of Wierzba III. Neurochem. Int..

[90] Saeed U., Durgadoss L., Valli R.K., Joshi D.C., Joshi P.G., Ravindranath V. (2008). Knockdown of Cytosolic Glutaredoxin 1 Leads to Loss of Mitochondrial Membrane Potential: Implication in Neurodegenerative Diseases. PLoS ONE.

[91] Ravindranath V. (2002). Neurolathyrism: Mitochondrial dysfunction in excitotoxicity mediated by L-β-oxalyl aminoalanine. Neurochem. Int..

[92] López-Martín M.C., Becana M., Romero L.C., Gotor C. (2008). Knocking out cytosolic cysteine synthesis compromises the antioxidant capacity of the cytosol to maintain discrete concentrations of hydrogen peroxide in arabidopsis. Plant Physiol..

[93] Hailu D., Abera S., Abera T. (2015). Effects of Processing on Nutritional Composition and Anti-Nutritional Factors of Grass pea (*Lathyrus sativus* L.): A review. Food Sci. Qual. Manag..

[94] Getahun H., Lambein F., Vanhoorne M., Stuyft P.V.D. (2005). Neurolathyrism risk depends on type of grass pea preparation and on mixing with cereals and antioxidants. Trop. Med. Int. Health.

[95] Tadele D., Alemu Y., Nigusie D., Peters K.J. (2003). Evaluation of Processing Methods on the Feeding Value of Grass Pea to Broilers. Int. J. Poult. Sci..

[96] Akalu G., Johansson G., Nair B.M. (1998). Effect of processing on the content of β-N-oxalyl-α, β-diaminopropionic acid (gb-ODAP) in grass pea (*Lathyrus sativus*) seeds and flour as determined by flow injection analysis. Food Chem..

[97] Buta M.B., Emire S.A., Posten C., Andrée S., Greiner R. (2019). Reduction of β-ODAP and IP6 contents in Lathyrus sativus L. seed by high hydrostatic pressure. Food Research International.

[98] Barik D.P., Mohapatra U., Chand P.K. (2005). Transgenic grasspea (*Lathyrus sativus* L.): Factors influencing Agrobacterium-mediated transformation and regeneration. Plant Cell Rep..

[99] Barna K.S., Mehta S.L. (1995). Genetic Transformation and Somatic Embryogenesis in *Lathyrus sativus*. J. Plant Biochem. Biotechnol..

[100] Girma D. (2010). Ethiopian Grass Pea (Lathyrus sativus L.) Started the Genomics Era: Transient Genetic Transformation of Grass Pea.

[101] Krishnan H.B. (2005). Engineering soybean for enhanced sulfur amino acid content. Crop Sci..

[102] Kortt A.A., Caldwell J.B., Lilley G.G., Higgins T.J.V. (1991). Amino acid and cDNA sequences of a methionine-rich 2S protein from sunflower seed (*Helianthus annuus* L.). Eur. J. Biochem..

[103] Kirihara J.A., Petri J.B., Messing J. (1988). Isolation and sequence of a gene encoding a methionine-rich 10-kDa zein protein from maize. Gene.

[104] Chui C.F., Falco S.C. (1995). A new methionine-rich seed storage protein from maize. Plant Physiol..

[105] Planta J., Xiang X., Leustek T., Messing J. (2017). Engineering sulfur storage in maize seed proteins without apparent yield loss. Proc. Natl. Acad. Sci. USA.

[106] Dinkins R.D., Reddy M.S.S., Meurer C.A., Yan B., Trick H., Thibaud-Nissen F., Finer J.J., Parrott W.A., Collins G.B. (2001). Increased sulfur amino acids in soybean plants overexpressing the maize 15 kDa zein protein. In Vitr Cell. Dev. Biol.-Plant.

[107] Kim W.S., Krishnan H.B. (2003). Allelic variation and differential expression of methionine-rich δ-zeins in maize inbred lines B73 and W23a1. Planta.

[108] Guo C., Liu X., Chen L., Cai Y., Yao W., Yuan S., Wu C., Han T., Sun S., Hou W. (2020). Elevated methionine content in soybean seed by overexpressing maize β-zein protein. Oil Crop Sci..

[109] Kim W.-S., Sun-Hyung J., Oehrle N.W., Jez J.M., Krishnan H.B. (2020). Overexpression of ATP sulfurylase improves the sulfur amino acid content, enhances the accumulation of Bowman–Birk protease inhibitor and suppresses the accumulation of the β-subunit of β-conglycinin in soybean seeds. Sci. Rep..

[110] Kim W.-S., Chronis D., Juergens M., Schroeder A.C., Hyun S.W., Jez J.M., Krishnan H.B. (2011). Transgenic soybean plants overexpressing O-acetylserine sulfhydrylase accumulate enhanced levels of cysteine and Bowman–Birk protease inhibitor in seeds. Planta.

[111] Avraham T., Badani H., Galili S., Amir R. (2005). Enhanced levels of methionine and cysteine in transgenic alfalfa (*Medicago sativa* L.) plants over-expressing the Arabidopsis cystathionine γ-synthase gene. Plant Biotechnol. J..

[112] Endo M., Mikami M., Toki S. (2016). Biallelic gene targeting in rice. Plant Physiol..

[113] Hilscher J., Bürstmayr H., Stoger E. (2017). Targeted modification of plant genomes for precision crop breeding. Biotechnol. J..

[114] Friedrichs S., Takasu Y., Kearns P., Dagallier B., Oshima R., Schofield J., Moreddu C. (2019). An overview of regulatory approaches to genome editing in agriculture. Biotechnol. Res. Innov..

[115] Zannoni L. (2019). Evolving Regulatory Landscape for Genome-Edited Plants. CRISPR J..

[116] Zhang D., Hussain A., Manghwar H., Xie K., Xie S., Zhao S., Larkin R.M., Qing P., Jin S., Ding F. (2020). Genome editing with the CRISPR-Cas system: An art, ethics and global regulatory perspective. Plant Biotechnol. J..

[117] Emmrich P.M., Sarkar A., Njaci I., Kaithakottil G.G., Ellis N., Moore C., Edwards A., Heavens D., Waite D., Cheema J. (2020). A draft genome of grass pea (*Lathyrus sativus*), a resilient diploid legume. BioRxiv.

[118] Friedrichs S., Takasu Y., Kearns P., Dagallier B., Oshima R., Schofield J., Moreddu C. (2019). Meeting report of the OECD conference on “genome editing: Applications in agriculture—Implications for health, environment and regulation”. Transgenic Res..

[119] Chilcoat D., Liu Z.B., Sander J. (2017). Use of CRISPR/Cas9 for crop improvement in maize and soybean. Prog. Mol. Biol. Transl. Sci..

[120] Fiaz S., Ahmad S., Noor M.A., Wang X., Younas A., Riaz A., Riaz A., Ali F. (2019). Applications of the CRISPR/Cas9 system for rice grain quality improvement: Perspectives and opportunities. Int. J. Mol. Sci..

[121] Wang H., Wu Y., Zhang Y., Yang J., Fan W., Zhang H., Zhao S., Yuan L., Zhang P. (2019). CRISPR/Cas9-Based Mutagenesis of Starch Biosynthetic Genes in Sweet Potato (Ipomoea Batatas) for the Improvement of Starch Quality. Int. J. Mol. Sci..

[122] Tuncel A., Corbin K.R., Ahn-Jarvis J., Harris S., Hawkins E., Smedley M.A., Harwood W., Warren F.J., Patron N.J., Smith A.M. (2019). Cas9-mediated mutagenesis of potato starch-branching enzymes generates a range of tuber starch phenotypes. Plant Biotechnol. J..

[123] Li J., Jiao G., Sun Y., Chen J., Zhong Y., Yan L., Jiang D., Ma Y., Xia L. (2021). Modification of starch composition, structure and properties through editing of *TaSBEIIa* in both winter and spring wheat varieties by CRISPR/Cas9. Plant Biotechnol. J..

[124] Nieves-Cordones M., Mohamed S., Tanoi K., Kobayashi N.I., Takagi K., Vernet A., Guiderdoni E., Périn C., Sentenac H., Véry A. (2017). Production of low-Cs^+^ rice plants by inactivation of the K^+^ transporter OsHAK1 with the CRISPR-Cas system. Plant J..

[125] Gomez M.A., Berkoff K.C., Gill B.K., Iavarone A.T., Lieberman S.E., Ma J.M., Schultink A., Karavolias N.G., Wyman S.K., Chauhan R.D. (2023). CRISPR-Cas9-mediated knockout of CYP79D1 and CYP79D2 in cassava attenuates toxic cyanogen production. Front. Plant Sci..

[126] Song J.H., Shin G., Kim H.J., Lee S.B., Moon J.Y., Jeong J.C., Choi H.-K., Kim I.A., Song H.J., Kim C.Y. (2022). Mutation of *GmIPK1* gene using CRISPR/Cas9 reduced phytic acid content in soybean seeds. Int. J. Mol. Sci..

[127] Sashidhar N., Harloff H.J., Potgieter L., Jung C. (2020). Gene editing of three *BnITPK* genes in tetraploid oilseed rape leads to significant reduction of phytic acid in seeds. Plant Biotechnol. J..

[128] Raffan S., Sparks C., Huttly A., Hyde L., Martignago D., Mead A., Hanley S.J., Wilkinson P.A., Barker G., Edwards K.J. (2021). Wheat with greatly reduced accumulation of free asparagine in the grain, produced by CRISPR/Cas9 editing of asparagine synthetase gene *TaASN2*. Plant Biotechnol. J..

[129] Kenar J.A. (2006). Reaction chemistry of gossypol and its derivatives. J. Am. Oil Chem. Soc..

[130] Lin J.-L., Fang X., Li J.-X., Chen Z.-W., Wu W.-K., Guo X.-X., Liu N.-J., Huang J.-F., Chen F.-Y., Wang L.-J. (2023). Dirigent gene editing of gossypol enantiomers for toxicity-depleted cotton seeds. Nat. Plants.

[131] Schachtsiek J., Stehle F. (2019). Nicotine-free, nontransgenic tobacco (*Nicotiana tabacum* L.) edited by CRISPR-Cas9. Plant Biotechnol. J..

[132] Zakaria M., Schemmerling B., Ober D. (2021). CRISPR/Cas9-mediated genome editing in comfrey (*Symphytum officinale*) hairy roots results in the complete eradication of pyrrolizidine alkaloids. Molecules.

[133] Ma J., Sun S., Whelan J., Shou H. (2021). CRISPR/Cas9-Mediated Knockout of *GmFATB1* Significantly Reduced the Amount of Saturated Fatty Acids in Soybean Seeds. Int. J. Mol. Sci..

[134] Wang Z., Shea Z., Rosso L., Shang C., Li J., Bewick P., Li Q., Zhao B., Zhang B. (2023). Development of new mutant alleles and markers for KTI1 and KTI3 via CRISPR/Cas9-mediated mutagenesis to reduce trypsin inhibitor content and activity in soybean seeds. Front. Plant Sci..

[135] Rajarammohan S., Kaur L., Verma A., Singh D., Mantri S., Roy J.K., Sharma T.R., Pareek A., Kandoth P.K. (2023). Genome sequencing and assembly of *Lathyrus sativus*—A nutrient-rich hardy legume crop. Sci. Data.

[136] Riepe M., Spencer P.S., Lambein F., Ludolph A.C., Allen C.N. (1995). In vitro toxicological investigations of isoxazolinone amino acids of *Lathyrus sativus*. Nat. Toxins.

[137] Saha T., Shee R., Sahid S., Shee D., Roy C., Sharma R., Pandey A., Paul S., Datta R. (2023). Designer grass pea for transgene-free minimal neurotoxin-containing seeds with CRISPR-Cas9. bioRxiv.

[138] Verma A., Kaur L., Kaur N., Bhardwaj A., Pandey A.K., Kandoth P.K. (2023). An efficient hairy root system for genome editing of a β-ODAP pathway gene in *Lathyrus sativus*. bioRxiv.

[139] Papadopoulos J.S., Agarwala R. (2007). COBALT: Constraint-based alignment tool for multiple protein sequences. Bioinformatics.

[140] Tamura K., Stecher G., Kumar S. (2021). MEGA11: Molecular Evolutionary Genetics Analysis Version 11. Mol. Biol. Evol..

[141] Letunic I., Bork P. (2021). Interactive Tree Of Life (iTOL) v5: An online tool for phylogenetic tree display and annotation. Nucleic Acids Res..

